# An Unforeseen Complication: Intestinal Prolapse After Total Abdominal Hysterectomy

**DOI:** 10.7759/cureus.53922

**Published:** 2024-02-09

**Authors:** Aditi Singh Thakur, Surekha Tayade, Vishnu K Gupta, Aishwarya Gupta, Nitish Batra

**Affiliations:** 1 Obstetrics and Gynecology, Datta Meghe Institute of Higher Education & Research, Wardha, IND; 2 General and Colorectal Surgery, VY Hospital, Raipur, IND; 3 Internal Medicine, Datta Meghe Institute of Higher Education & Research, Wardha, IND

**Keywords:** vaginal cuff dehiscence, case report, surgical complication, total abdominal hysterectomy, intestinal prolapse

## Abstract

After total abdominal hysterectomy (TAH), intestinal prolapse is uncommon. We report an instance of a 48-year-old woman who had TAH and then intestinal prolapse. Two weeks after the operation, symptoms started to show up, and the vaginal vault developed a bulging bulge. The problem was satisfactorily treated with an urgent laparotomy. The significance of being vigilant for unusual complications following TAH is shown by this example.

## Introduction

Extra-fascial hysterectomy is a popular term used to describe total abdominal hysterectomy (TAH) [1]. Total abdominal hysterectomy (TAH) is a surgical procedure used to treat various gynecologic conditions, providing a definitive solution for patients seeking relief. Gynecologists need to be proficient in TAH, which is a procedure that removes the uterus. Urinary tract trauma, damage to the intestinal tract, and bladder injury are possible after TAH. It's crucial to follow the proper release layer protocol and confirm that "the uterus was truly naturally removed" to prevent surgical problems [1]. The dehiscence and evisceration of vaginal cuffs were initially documented in 1864 [1]. The two occurrences are uncommon. Organs that are intraperitoneally expelled through a separated incision are referred to as vaginal cuff dehiscence accompanied by evisceration. The frequency was between 0.14 and 4.1% [2]. Following pelvic surgery, particularly a hysterectomy, vaginal cuff dehiscence, and evisceration are major postoperative complications that call for immediate resuscitation and surgical intervention. Following a hysterectomy, vaginal evisceration can happen at any moment; cases have been documented as soon as three days and as late as thirty years following surgery [3]. The significance of recognizing and comprehensively addressing such infrequent complications cannot be understated, as their prompt identification and management are imperative to avert potential adverse outcomes. Despite its rarity, the occurrence of intestinal prolapse post-TAH necessitates a more profound understanding to refine surgical techniques, optimize postoperative care, and potentially prevent similar complications in future surgical cases.

Additionally, elevated pressure within the abdomen in postmenopausal women and sexual activity in premenopausal patients were the most common triggering events. Furthermore, the most often eviscerated organ was the small bowel. The majority of the patients had a bulging mass, pelvic discomfort, or vaginal bleeding when they first arrived [4]. This case report centers on a 48-year-old female patient who underwent an uncomplicated TAH for symptomatic uterine fibroids. Approximately two weeks following discharge, the patient presented with subtle yet progressive symptoms, eventually leading to the diagnosis of intestinal prolapse. The intricate clinical course, successful surgical management, and subsequent recovery of the patient serve as a focal point for elucidating the complexities associated with this atypical complication.

The report aims to comprehensively explore the patient's clinical journey, including symptom onset, diagnostic challenges, surgical intervention, postoperative care, and implications for healthcare practice. By delving into the intricacies of this rare occurrence, this report seeks to contribute to the medical understanding of uncommon complications post-TAH, emphasizing the necessity for heightened vigilance, meticulous surgical techniques, and tailored postoperative care strategies.

## Case presentation

A 48-year-old woman, who had a history of three pregnancies and three deliveries, sought urgent care at the emergency department due to distressing symptoms. She had recently undergone a total abdominal hysterectomy 11 weeks before her presentation, a procedure aimed at addressing problematic uterine fibroids. The patient, who meticulously adhered to the prescribed postoperative instructions, suddenly experienced a distressing episode while attempting a bowel movement. During this moment, she observed vaginal bleeding and had a startling sensation of something being expelled from her vagina. These alarming symptoms prompted her immediate visit to the emergency room. Besides this acute event, the patient had been grappling with escalating pelvic pressure over the past few weeks, particularly noticeable during bowel movements. Her vital signs were taken upon her arrival, which showed that her blood pressure was 104/65 mmHg, her pulse rate was 80 bpm, her temperature was 37.8 °C, her rate of respiration was 19/minute, and her oxygen saturation was 96% on room air. A physical examination revealed no obvious signs of peritoneal irritation, such as rebound, stiffness, or voluntary guarding, but relatively moderate to severe suprapubic pain.

Further investigation via a pelvic examination unveiled a distressing finding - a portion of the small intestine is protruding from the upper part of her vaginal area. This concerning discovery prompted an immediate consultation with the general surgery team. Given the possibility of severe conditions such as bowel ischemia or early perforation, an urgent and comprehensive workup was recommended.

Subsequent laboratory analysis unveiled indications of sepsis, necessitating the prompt initiation of broad-spectrum antibiotics, as shown in Table 1. A total dose of 4.5 grams of piperacillin-tazobactam was administered to combat the suspected infection.

**Table 1:** List of diagnostic tests conducted on this patient

Investigations	Observed value	Expected value
Haemoglobin (gm%)	12.4	12-16
White blood cells (cu..mm)	18300	4000-11000
HBA1c	4.4	≤ 5.6
Serum urea (mg/dL)	24	6-24
Serum creatinine (mg/dL)	0.8	0.7-1.2
Serum Sodium (mEq/L)	139	131-145
Serum potassium (mmol/L)	4.4	3.6-5.2
Thyroid-stimulating hormone (mlU/L)	2.2	0.5-5.0
Free T3 (pg/mL)	3.2	2.3-4.1
Free T4 (pg/mL)	11.6	9.0-17.0
Albumin	3.9 g/dl	3.5-5.0 g/dl
Aspartate aminotransferase	43 U/L	<50 U/L
Alanine aminotransferase	29 U/L	17-59 U/L
Total bilirubin	0.7 mg/dl	0.2-1.3 mg/dl
International normalized ratio	0.9	0.8-1.1
Prothrombin time	11.8	11.9
Activated partial thromboplastin time (APTT)	30.6	29.5
C-reactive protein	10.7	<0.3mg/dl

**Figure 1:**
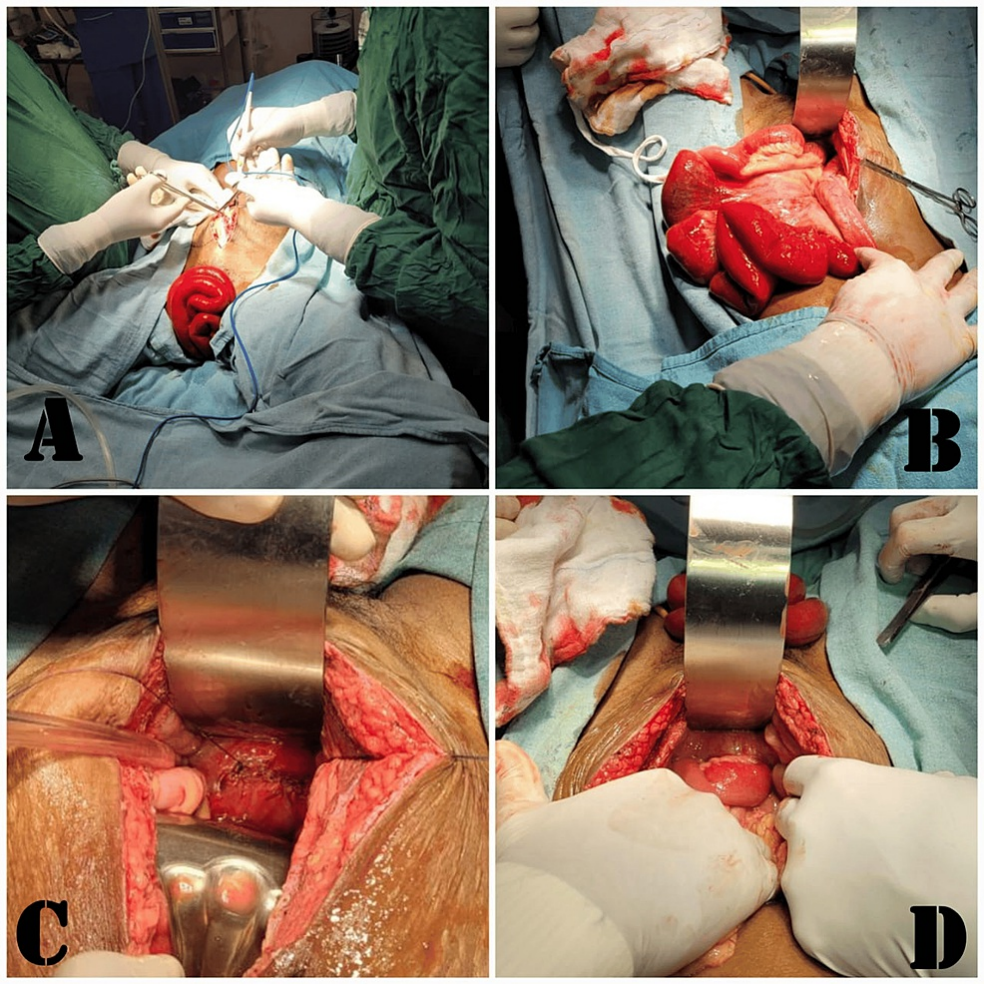
A) Bulging of a small intestinal segment from the vagina, B) careful manual reduction of the eviscerated bowel, C) handling of the vaginal cuff dehiscence, and D) verification of hemostasis before the conclusive closure

Urgent surgical intervention via laparotomy under general anesthesia was swiftly arranged. In the operating room, a careful examination revealed complete vaginal cuff dehiscence with no evident signs of active infection. However, there were noticeable weaknesses and friability in the tissues between the anterior vaginal wall and the bladder wall. A critical intraoperative finding was the identification of a segment of the small intestine herniating through the dehisced vaginal cuff, causing the prolapse. The surgical approach involved a meticulous manual reduction of the eviscerated bowel and precise management of the vaginal cuff dehiscence. The procedure included the removal of granulation tissue at the vaginal cuff edge and a meticulous closure using simple interrupted sutures through a vaginal approach. Careful reduction of the prolapsed bowel was performed. Figure 1 illustrates the intraoperative observations.

Postoperatively, the patient received a two-day course of 4.5 grams of piperacillin-tazobactam to mitigate the risk of infection. Her recovery was closely monitored, focusing on detecting any signs of infection, restoring proper bowel function, and effectively managing pain. Followup assessments indicated a favorable recovery trajectory, with the resolution of the initial distressing symptoms and successful management of the emergent situation through surgical intervention.

## Discussion

Intestinal prolapse following a total abdominal hysterectomy (TAH) represents an exceptionally uncommon complication, with sparse documentation within the medical literature [5]. This case serves as a poignant reminder of the necessity for clinicians to remain vigilant regarding even the rarest of postoperative complications, particularly those affecting the genitourinary system. Despite its infrequency, the potential severity and urgency of intervention mandate a thorough comprehension of the predisposing factors and management strategies associated with this condition.

Multiple factors may contribute to the development of intestinal prolapse following TAH. One such factor is the heightened intra-abdominal pressure experienced during the initial postoperative phase. Activities such as lifting heavy objects, prolonged straining during bowel movements, or persistent coughing may collectively exert undue pressure on the abdominal cavity, potentially predisposing individuals to the development of intestinal prolapse [6]. Furthermore, technical issues or inadequate healing related to the closure of the vaginal cuff after hysterectomy may render the patient susceptible to vaginal cuff dehiscence, thereby facilitating the herniation of intestinal structures.

Recognizing intestinal prolapse as a plausible postoperative complication is paramount to ensuring timely diagnosis and intervention. Close monitoring during the postoperative recovery period, coupled with comprehensive patient education regarding the avoidance of activities that may exacerbate intra-abdominal pressure, can serve as crucial preventive measures. Moreover, meticulous attention to surgical techniques during vaginal cuff closure is imperative in minimizing the risk of dehiscence and subsequent complications [7, 8].

In instances where intestinal prolapse does manifest, urgent surgical intervention becomes imperative. Timely reduction of the prolapsed bowel followed by meticulous repair of the vaginal cuff is paramount to achieving a favorable postoperative outcome. Subsequent postoperative care entails vigilant monitoring for signs of infection, restoration of bowel function, and effective pain management to facilitate patient recovery and alleviate discomfort [9].

While this case underscores successful management, further research endeavors are warranted to elucidate preventive strategies and refine surgical techniques. Prospective studies aimed at evaluating optimal methods for vaginal cuff closure, strategies for mitigating intra-abdominal pressure post-hysterectomy, and long-term outcomes in analogous cases would undoubtedly yield invaluable insights into minimizing the incidence of such rare complications [10]. In summation, ongoing research initiatives and clinical diligence are indispensable in addressing and managing intestinal prolapse following TAH, thereby ensuring optimal patient outcomes and quality of care.

## Conclusions

This case of intestinal prolapse post-total abdominal hysterectomy emphasizes the need for careful monitoring and understanding of postoperative risks. The patient's experience, successful surgery, and recovery offer valuable insights into this rare issue. Timely recognition and intervention were crucial for a positive outcome, underscoring the importance of prompt action in managing such complications. This case encourages further research to improve surgical techniques and postoperative care to prevent similar occurrences after hysterectomy. Enhanced awareness and ongoing studies are vital in improving patient care and outcomes following gynecologic surgeries, particularly in addressing uncommon complications like intestinal prolapse.
